# Hijacked then lost in translation: the plight of the recombinant host cell in membrane protein structural biology projects

**DOI:** 10.1016/j.sbi.2015.04.003

**Published:** 2015-06

**Authors:** Roslyn M Bill, Tobias von der Haar

**Affiliations:** 1 School of Life & Health Sciences, Aston University, Aston Triangle, Birmingham B4 7ET, UK; 2 Kent Fungal Group, School of Biosciences, University of Kent, Canterbury CT2 7NJ, UK

## Abstract

Membrane protein structural biology is critically dependent upon the supply of high-quality protein. Over the last few years, the value of crystallising biochemically characterised, recombinant targets that incorporate stabilising mutations has been established. Nonetheless, obtaining sufficient yields of many recombinant membrane proteins is still a major challenge. Solutions are now emerging based on an improved understanding of recombinant host cells; as a ‘cell factory’ each cell is tasked with managing limited resources to simultaneously balance its own growth demands with those imposed by an expression plasmid. This review examines emerging insights into the role of translation and protein folding in defining high-yielding recombinant membrane protein production in a range of host cells.

## Highlights

• Membrane protein structural biologists need high-quality protein for crystallisation.
• Recombinant proteins are central to the structural biology supply chain.
• Understanding quality control in protein production is an emerging trend.
• The roles of translation and protein folding in the host cell are examined.

## Graphical abstract

**Figure:**
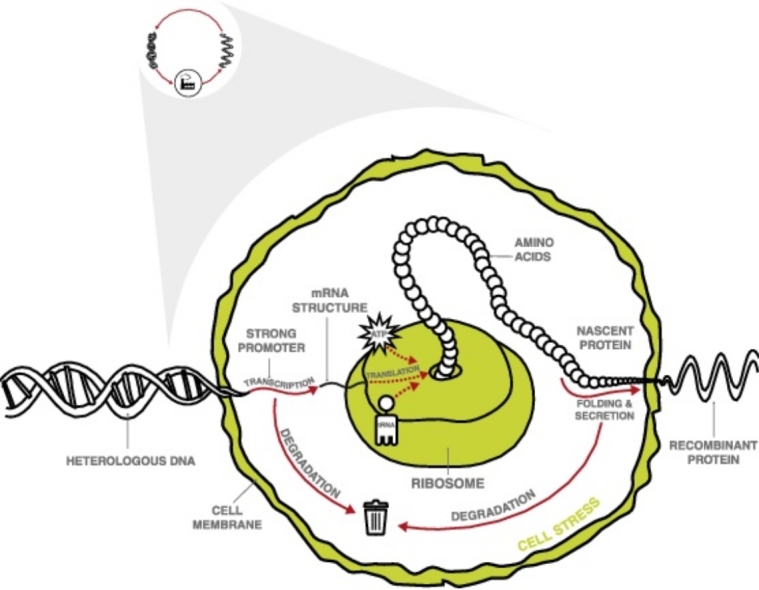

**Current Opinion in Structural Biology** 2015, **32**:147–155

This review comes from a themed issue on **New constructs and expressions of proteins**

Edited by **Imre Berger** and **Roslyn M Bill**

For a complete overview see the Issue and the Editorial

Available online 1st June 2015

**http://dx.doi.org/10.1016/j.sbi.2015.04.003**

0959-440X/© 2015 The Authors. Published by Elsevier Ltd. This is an open access article under the CC BY license (http://creativecommons.org/licenses/by/4.0/).

## Recombinant proteins are essential to progress in modern structural biology

**Figure 1:**
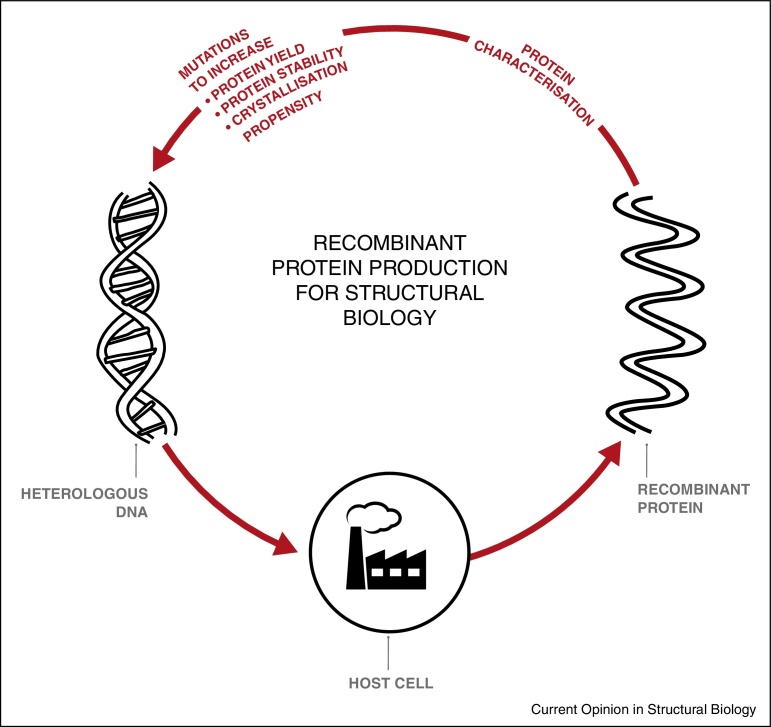
Recombinant protein production for structural biology. Heterologous DNA is introduced into a host cell with the aim of producing a recombinant protein. This typically necessitates mutagenesis of the expression construct to increase protein yield plus the incorporation of additional mutations to stabilise the resultant protein so it is more likely to crystallise. These manipulations can be done pre-translationally by mutating the gene sequence or fusing it with a stabilising partner. Alternatively, the protein can be engineered post-translationally. In many cases, many tens or even hundreds of constructs are examined before proceeding to structural studies.

**Figure 2:**
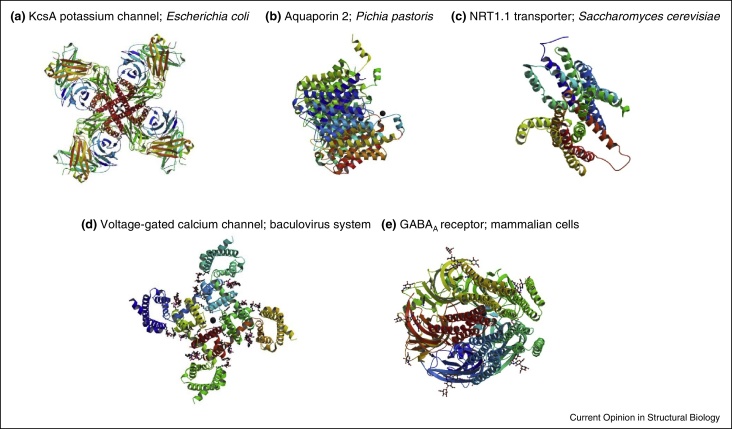
Examples of recent structures of recombinant membrane proteins. The name of the protein and the host cell used in its recombinant production are given. Structural images were downloaded from the PDB website (http://www.pdb.org/pdb/home/home.do) on 30th March 2015; protein chains are coloured from the amino-terminus to the carboxy-terminus using a spectral colour gradient. **(a)** The structure of the KcsA potassium channel (PDB code: 2JK5) was solved to 2.4 Å using a truncated protein produced in *E. coli* in which thirty-five residues of KcsA had been removed with chymotrypsin. **(b)** The structure of the aquaporin 2 water channel was solved to 2.75 Å (PDB code: 4NEF) using recombinant protein produced in *P. pastoris* following codon optimisation of the corresponding gene sequence. **(c)** The structure of the NRT1.1 nitrate transporter at 3.7 Å (PDB code: 4CL4) was solved using a fusion protein with carboxy-terminal GFP and hexahistidine tags that had been produced in *S. cerevisiae*. **(d)** The structure of a voltage-gated calcium channel at 2.75 Å (PDB code: 4MS2) was solved after six mutant forms of the protein were produced using the baculovirus system. **(e)** The 3 Å crystal structure of the GABA_A_ receptor (PDB code: 4COF) was solved after approximately one-hundred construct variants of the full-length human β3 subunit were evaluated in a mammalian cell expression system.

Progress in soluble protein structural biology continues at a ‘breathless pace’ [1], while success in the membrane protein field lags behind [2]. However, recent advances in experimental approaches [3] mean that even the most recalcitrant membrane proteins are now tenable structural biology targets; this requires sufficient, stable recombinant protein for biochemical characterisation and crystallisation trials. A widely used strategy is illustrated in Figure 1, where the chosen host cell is regarded as a ‘cell factory’ that can be used to produce the target protein. This typically necessitates mutagenesis of the expression construct to increase protein yield [4] plus the incorporation of additional mutations to stabilise the resultant protein so it is more likely to crystallise [3]. These manipulations can be done pre-translationally by mutating the gene sequence (e.g. [5]) or fusing it with a stabilising partner (e.g. [6^•^]). Alternatively, the protein can be engineered post-translationally by deglycosylation, proteolysis or by making other chemical modifications [7]. The 3 Å crystal structure of the GABA_A_ receptor (Figure 2) was solved after approximately one-hundred construct variants of the full-length human β3 subunit were evaluated (in a stable mammalian cell-line [8]); the consequences were examined of N-linked glycosylation site removal, mutation of cysteine residues, amino-terminal and carboxy-terminal truncations, truncations in the intracellular loop connecting transmembrane helices 3 and 4 and introduction of T4 lysozyme to minimise loop flexibility [9^•^]. The structure of the TRPV1 channel at 3.4 Å was possible because recent developments in electron cryo-microscopy meant that absolute conformational homogeneity was not required and only small amounts of protein were needed; consequently a less extensive mutational strategy was necessary [10]. Even in this case, however, the recombinant TRPV1 variant (produced using a modified baculovirus system) was composed of amino acids 110–603 and 627–764, demonstrating the importance of prior biochemical characterisation [10]. Similar methodological approaches have been used for proteins produced in *Escherichia coli* [e.g. 11], *Saccharomyces cerevisiae* [e.g. 12] and *Pichia pastoris* [e.g. 13] (see also Figure 2, Table 1).

## Understanding the host cell provides new opportunities to improve functional yields

**Figure 3:**
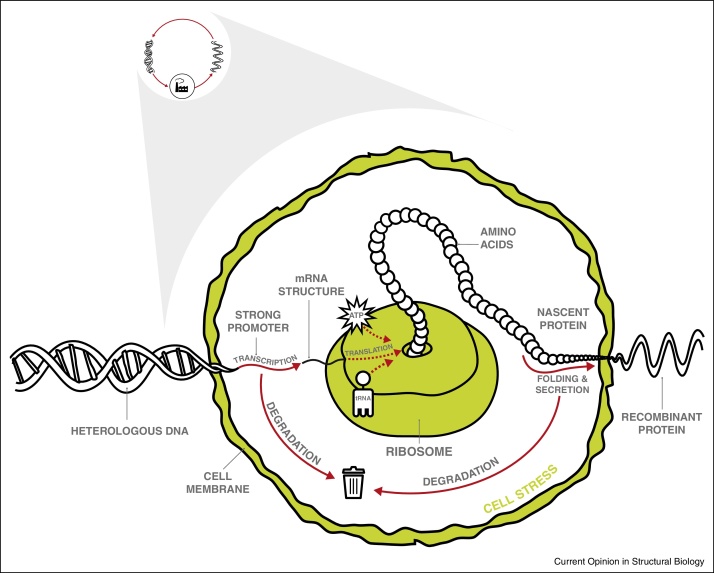
Insights into the workings of a recombinant host cell factory. A host–cell-centric approach to producing recombinant proteins for structural analysis focuses on maximising the functional yield of every cell; in this case, the philosophy is to understand the workings of the ‘cell factory’ shown in Figure 1. Any recombinant host cell must simultaneously balance its requirements for cellular growth with the metabolic burden imposed by the expression plasmid, where transcription is typically under the control of a strong promoter (see Table 1) although this may be countered by high rates of mRNA degradation. Factors affecting the successful production of a recombinant protein include the availability of energy (in the form of ATP and/or GTP), tRNAs, ribosomes, mRNA structure, the integrity of the folding and secretory pathways and cell stress responses. For membrane proteins, translation and protein folding are critical parameters; their modulation may help to further improve host cells.

A complementary, host–cell-centric approach to producing recombinant proteins for structural analysis focuses on maximising the functional yield of every cell; in this case, the philosophy is to understand the workings of the ‘cell factory’ (Figure 3). Any recombinant host cell must simultaneously balance its requirements for cellular growth with the metabolic burden imposed by the expression plasmid. Consequently, if it were possible to uncouple growth from recombinant synthesis in a host cell that remained metabolically capable of transcription and translation, this might enable metabolic fluxes to be entirely diverted to the production of a recombinant protein [14]. This concept has been demonstrated in *E. coli* to produce soluble chloramphenicol acetyltransferase to more than 40% of total cell protein [15], but has not been widely adopted by structural biologists. A concept that has gained more traction focuses on the idea that the modulation of translation and protein folding may help to further improve host cells [16]. This review examines emerging insights into the role of these dependent pathways in defining high-yielding recombinant membrane protein production experiments.

## Post-transcriptional bottlenecks in recombinant protein production

Many commonly used recombinant expression systems (Table 1) use strong promoters to drive high rates of mRNA synthesis (although this may be countered by high rates of mRNA degradation [17]). In the *E. coli* Walker strains C41(DE3) and C43(DE3) [18], acquired mutations were found to lower the efficiency of the T7 promoter leading to improved yields of membrane proteins for some but not all targets [19]. Similarly, in the *E. coli* MemStar system, the activity of T7 RNA polymerase was found to be modulated [20]. In a separate study, a series of evolved *E. coli* strains that had been selected based on their resistance to erythromycin were found to have a mutation in the *hns* gene, which has a role in transcriptional silencing [21]. These examples suggest that a common theme of prokaryotic strains selected for their high-yielding properties is a tendency to rebalance mRNA and protein synthesis rates, although the detail of how this is achieved is not yet understood.

A recent analysis of functional yields (by radioligand binding) and total yields (by immunoblotting) of recombinant angiotensin II type 1 receptor produced in insect cells showed that the majority of the protein was non-functional. When the same protein was produced in a stable, inducible HEK293 cell-line that had been selected by fluorescence-activated cell sorting to identify a high-yielding clone, a similar total yield was obtained, but the majority of the protein was now functional [22^••^]. Several factors may account for this observation: first, the strong polyhedrin promoter (polyhedrin mRNA accounts for 20% of polyadenylated mRNA in the cell [23]) is likely to produce very high levels of mRNA that overwhelm post-transcriptional pathways; second, viral-based expression systems impair the secretory pathway of a host cell [24], which will negatively impact on the ability of the nascent protein to fold.

Recovering functional protein from recombinant host cells is critically dependent upon the ability of the host to synthesise an authentically folded protein, which in turn depends on the proper functioning of its secretory pathway [25]. Recombinant eukaryotic proteins are often produced in low yields or are misfolded in prokaryotic host cells [26]. One explanation for this is that polypeptide synthesis rates are faster in prokaryotes (10–20 amino acids *per* s) than in eukaryotes (3–8 amino acids *per* s) [27^••^]. A popular strategy to mitigate this has been to decrease culture temperature, but many cellular functions that impact yield (e.g. transcription, translation, folding rates, membrane composition) are also affected by low temperature stress [28], which means that yield increases do not always transpire. Experiments in *E. coli* cells with mutant ribosomes (whose translation speed can be modulated) showed that reducing polypeptide elongation rates enhanced the folding efficiency of soluble firefly luciferase, the cycle 3 mutant of *Aequorea victoria* green fluorescent protein (GFP) and *S. cerevisiae* Cdc13p [27]. This suggests that protein folding requires slow translation rates in eukaryotes; in contrast, folding in bacteria is uncoupled from protein synthesis. Notably, in these experiments, decreasing translation rates in *E. coli* did not result in endogenous protein misfolding or the activation of a bacterial stress response [27].

## Bottlenecks in translation

Translation is a highly regulated process that requires interactions between mRNA, ribosomes and a large number of other molecules in a complex, but optimised network that has been well-described for prokaryotes [29] and eukaryotes [30]. In eukaryotic cells, a key player in translational regulation is the mammalian target of rapamycin (mTOR) complex, which controls ribosome and tRNA biogenesis, translation initiation and entry into quiescence (G0 phase) [31]; mTOR is a central regulator of cell metabolism, growth, proliferation and survival, demonstrating the essential link between translation and host cell physiology.

The translational machinery of a cell responds to its growth rate, which is strongly affected by nutrient availability. Changes in nutrient source were recently shown to significantly reprogramme the transcriptome of *P. pastoris* under growth conditions relevant to recombinant protein production [32]. The main response was found to be transcriptional, while translational regulation was global rather than transcript-specific. When *P. pastoris* cells were cultured in methanol, a high proportion of the mRNA pool was associated with two or more ribosomes (and therefore deemed to be highly translated); methanol is typically used to induce protein production in this yeast species, which suggests that high recombinant protein yields may be associated with the slow growth rate observed under these conditions as well as promoter activity [33]. It has been known for decades that ribosomes from slow-growing cells have lower amino acid incorporation rates per second per ribosome than cells that are growing normally [34^••^]. Slowing translation speed may therefore enhance recombinant protein folding efficiency. In support of this, partial inhibition of translation in mammalian host cells (using the drug, emetine) was shown to result in a substantial reduction in the yield of misfolded *Pontellina plumata* GFP; emetine treatment also increased the functional recombinant yields of both GFP and a ΔF508 mutant of the cystic fibrosis transmembrane conductance regulator [35]. Lower translation speed has been postulated to directly lead to higher translational accuracy [36]. This is difficult to reconcile with current biochemical knowledge of translation but if the relationship does indeed exist, slower translation could be beneficial for the production of difficult-to-fold proteins that are particularly sensitive to translational errors.

The sequence of the mRNA transcript is also important in determining the rate and accuracy of protein translation [37]. It is established that individual species have a preference for certain of the 64 available codons over others, but the biological reason for this is unclear. One idea is that each codon has a different decoding time; ‘faster’ codons lead to a higher translation rate, which is more resource efficient [38]. Recently, ‘speed control signals’ have been proposed in the signal peptides of secreted proteins that delay translation to allow co-translational and post-translational translocation, protein processing and folding [39]. Codon usage bias was found be normal in open reading frames, but decreased in signal peptide coding regions [39]. This may have consequences for the optimal design of the heterologous DNA construct in Figures 1 and 3. Another idea is that different codons are read with different degrees of accuracy: when translational accuracy was included as a parameter in the design of expression constructs for a *Plasmodium* lysyl-tRNA synthetase in *E. coli*, proteolysis was reduced and solubility increased [40].

## Bottlenecks in folding and secretion

Translation, folding and secretion are interconnected pathways that draw on the same cellular resources. If the interconnections are not optimised in favour of the recombinant protein, then yields will be low (Figure 3). Most nascent proteins do not fold spontaneously in vivo, but require a network of chaperones that not only facilitate protein folding, but also perform quality control, ensuring that damaged or misfolded proteins are degraded or refolded. Many studies have focused on the co-expression or down-regulation of individual chaperones to improve recombinant protein yields [41], but a current trend is to exploit a host cell's global stress response to misfolded proteins to ensure the proper functioning of its secretory pathways; this approach is anticipated to be more effective in improving the quantity and quality of recombinant protein for structural studies [42^•^]. In eukaryotes, even moderate levels of stress cause a decrease in the rate of translation, the sequestering of mRNA into stress granules and the aggregation of proteins [43]. It still remains unclear how cells regulate this response, but the ability to influence the distribution of diverse chaperones in vivo may offer solutions to improving protein yields [44]. For example, a recent study of recombinant G protein-coupled receptor production in *S. cerevisiae* demonstrated that mislocalised proteins were associated with the endoplasmic reticulum chaperone, BiP [45].

The impact of two cellular responses is of particular interest: The unfolded protein response (UPR; influencing the early part of the secretory pathway) and the heat shock response (HSR; a response to cytosolic stress). Recombinant protein production is likely to affect ER homeostasis and therefore trigger a UPR, which in turn causes an increase in the folding capacity of a cell [25]; recently biosensors have been used to measure the UPR in mammalian cell-lines producing monoclonal antibodies [46]. During the UPR in yeast, about 5% of the genome is up-regulated (mirroring the situation in higher eukaryotes), ribosomal biogenesis and assembly are translationally repressed and mRNAs encoding the UPR transcription factor Hac1p, the ER-oxidoreductase Ero1p and the ER-associated protein degradation (ERAD) protein Der1p, are enriched in polysomal fractions, indicating translational up-regulation [47]. The HSR activates chaperones and the proteasome in order to relieve stress. HSR up-regulation has been used to increase recombinant yields of soluble α-amylase in *S. cerevisiae*, but did not increase the yield of a recombinant human insulin precursor [48]. It is now established that the UPR, HSR and other stress responses (such as the environmental stress response; ESR) overlap with each other, providing a hormetic benefit to cells in which mild stress enhances tolerance to future stressful stimuli [49] such as that imposed during recombinant protein production.

## Conclusions

The use of stabilising mutations to improve the crystallisation propensity of recombinant membrane proteins has resulted in major breakthroughs in modern structural biology [3]. Our emerging understanding of how the processes of translation and protein folding are affected in recombinant host cells now offers new, complementary opportunities to improve functional yields. However, the interlinked nature of transcription, translation, folding and secretion and their impact on cell physiology means that optimising host cells to be maximally productive with respect to functional recombinant protein yield is both intellectually and technically demanding. Table 1 suggests a dominant role for microbial host cells in membrane protein structural biology projects; since microbes are particularly amenable to genetic engineering, new insight may emerge from their use in the foreseeable future.

## Conflict of interest statement

Nothing declared.

## References and recommended reading

Papers of particular interest, published within the period of review, have been highlighted as:

• of special interest
•• of outstanding interest

## Figures and Tables

**Table 1:** Details of host cells used to produce recombinant α-helical transmembrane proteins for structures published in 2014 and 2015; representative structures are illustrated in Figure 2

Description	Host strain	Promoter	Protein produced (with PDB code in parentheses)
*Escherichia coli*			
The first choice host cell for many recombinant protein production experiments; functional yields may be low especially for eukaryotic targets [50] and expression is typically via episomal plasmids that are transiently transformed into the host cell	BL21 (DE3)	pT7*lac* is one of the strongest prokaryotic promoters [51]; induced with isopropyl β-D-1-thiogalactopyranoside (IPTG)	AF2299 CDP-alcohol phosphotransferase (4O6M), Atm1-type ABC exporter (4MRN and 4MYC), bacterial homologue of human ASBT (4N7W), bacterial homologue of the BEST1 Ca^2+^-activated Cl^−^ channel (4WD7), cytochrome b_561_ (4O6Y), insulin receptor transmembrane domain (2MFR), KirBac3.1 inward-rectifier potassium channel (4LP8), mitochondrial translocator protein (2MGY), neurotensin receptor 1 (4BUO), PepT_So_ oligopeptide-proton symporter (4TPH and 4UVM), prokaryote ligand-gated ion channel ELIC (4TWD), semisweet transporter (4QNC), translocator protein (4RYQ), translocator protein (4UC1), UbiA prenyltransferase (4TQ3),vascular endothelial growth factor receptor 2 transmembrane dimer (2M59), voltage-sensing domain of a voltage-sensitive phosphatase (4G7V), YetJ pH-sensitive calcium-leak channel (4PGR)
	BL21 (DE3) *ΔacrAB*		NADH transhydrogenase (4O93)
	C41(DE3) [17]		MgtE Mg^2+^ transporter (4U9L), Na_V_Ae1p voltage-gated sodium channel (4LTO), vitamin K epoxide reductase (3KP9), YidC_27-266_ insertase (3WO6)
	C43 (DE3) [17]		Pentameric ligand-gated ion channel GLIC (4TWD), Δ^14^ sterol reductase (4QUV)
	Rosetta (DE3)		α7 neuronal Ach receptor (2MAW)
	Novablue (DE3)	pT5*lac* is one of the strongest prokaryotic promoters [51]; induced with IPTG	KcsA potassium channel (2JK5; Figure 2a)
	C43 (DE3)	p*BAD* is typically induced by arabinose	CmeC bacterial multi-drug efflux transporter (4MT4), heterodimeric ABC exporter (4Q4H)
	MC1061		PnuC vitamin B3 transporter (4QTN), SLC11 (NRAMP) transition-metal ion transporter (4WGV)
	TOP10		Glutamate transporter homologue (4P19 and 4X2S)

*Pichia pastoris*			
Methylotrophic yeast noted for its ability to grow to very high cell densities [52]; expression is typically via stably-integrated expression cassettes [13]	GS115	p*AOX1* is a very strong promoter [13,33] typically induced with methanol	P-glycoprotein (4M1M)
	GS115 *aqy1Δ*		Aquaporin 2 (4NEF; Figure 2b)
	KM71H		Leukotriene LTC_4_ synthase (4JCZ)
	SMD1163; lacks proteinase A (Pep4) and B (Prb1) activity [13]		Bestrophin-1 Ca^2+^-activated Cl^−^ channel (4RDQ), P-glycoprotein homologue CmABCB1 (3WME), two-pore domain potassium channel K_2P_4.1 (4WFF)

*Saccharomyces cerevisiae*			
Yeast with a wide range of genetic resources that have enabled host engineering studies [26]; expression is typically via episomal plasmids that are transiently transformed into the host cell, but stable integration is sometimes used	FGY217; deletion of the *pep4* gene in this yeast strain inhibits Pep4 protease activity and reduces the levels of other vacuolar hydrolases [53]	p*GAL1* is induced with galactose	NRT1.1 nitrate transporter (4CL4; Figure 2c), TMEM16 Ca^2+^-activated lipid scramblase (4WIS)

*Insect cells*			
Widely used host [54], especially in the production of G protein-coupled receptors, although functional yields may be low [22]; expression occurs through the generation of viral particles that are used to infect insect cell cultures	*Spodoptera frugiperda*	The polyhedrin promoter is one of the strongest eukaryotic promoters [23]; it is constitutive	ASIC1 acid-sensing ion channel (4NTW), claudin-15 (4P79), GluA2 glutamate receptor (4U4G), GluN1a/GluN2B NMDA receptor (4EP5), GPR40 free fatty-acid receptor 1 (4PHU), Hv1 chimeric voltage-gated proton channel (3WKV), metabotropic glutamate receptor 1 (4OR2), metabotropic glutamate receptor 5 (4OO9), NRT1.1 nitrate transporter (4OH3), δ-opioid receptor (4N6H), P2Y_12_ receptor (4NTJ and 4PXZ), γ-secretase nicastrin extracellular domain (4R12)
	*Trichoplusia ni*		GLUT1 glucose transporter (4PYP), voltage-gated calcium channel (Ca_V_; 4MS2; Figure 2d)

*Mammalian cell culture*			
An authentic host for producing fully functional [55] mammalian proteins [56]; expression can be via transient transfection or stable integration	HEK293S GnTI^−^; N-acetylglucosaminyl transferase I-negative cells that are unable to synthesise complex N-glycans [57]; gene transduction of mammalian cells was baculovirus-mediated	The cytomegalovirus (CMV) promoter is strong in this host cell [58] and is constitutive	GluA2 glutamate receptor (4U2P and 4U5B), GluN1a/GluN2B NMDA receptor (4TLL)
	HEK293F	Chick actin promoter with a CMV enhancer [59]	γ-Secretase nicastrin extracellular domain (4UPC)
	HEK293T		GABA_A_ receptor (4COF; Figure 2e)
	T-REx-293 (a stable cell-line was generated)	CMV promoter	Serotonin 5-HT_3_ receptor (4PIR)
